# Natural Immunomodulators Treat the Cytokine Storm in SARS-CoV-2

**DOI:** 10.34172/apb.2023.006

**Published:** 2021-10-02

**Authors:** Heba Salah Abbas, Mona Mohame Abd-elhakeem, Rania Mostafa Abd El Galil, Omar Ahmed Reyad, Heba Ahmed Mohamed, Salma Emad Saber Ismail, Manal Ahmed Nabil

**Affiliations:** ^1^Microbiology Department, National Organization for Drug Control and Research(NODCAR), Egyptian Drug Authority, Giza, Egypt.; ^2^Biochemistry /Faculty of Art & science, Jazan University, Saudi Arabia.; ^3^Pharmaceutics Department, Faculty of Pharmacy, Misr University of Science and Technology.; ^4^High Institute of Public Health, Aexanderia, Egypt.; ^5^Master Student, Microbiology, Faculty of Science, Suez University, Egypt.; ^6^Faculty of Pharmacy, Ain Shams University, Egypt.; ^7^Department of Immunology & Allergy, Medical Research Institute, Alexandria University, Alexandria, Egypt.

**Keywords:** SARS-COV-2, Cytokine storm syndrome, Polyphenols, Zinc treatment, Lactoferrin, Medicinal plants and essential oils

## Abstract

Recently, the world has been dealing with a destructive global pandemic Coronavirus disease 2019 (COVID-19) infection, since 2020; there were millions of infections and hundreds of thousands of deaths worldwide. With sequencing generations of the virus, around 60% are expected to become infected during the pandemic. Unfortunately, no drug or vaccine has been approved because no real evidence from clinical trials in treatment was reached. According to current thinking, severe acute respiratory syndrome coronavirus 2 (SARS-CoV-2) mortality is caused by a cytokine storm syndrome in patients with hyper-inflammatory conditions, resulting in acute respiratory distress and finally death. In this review, we discuss the various types of natural immune-modulatory agents and their role in the management of SARS-CoV-2, and cytokine storm syndrome. For example, Polyphenols as natural products can block the binding of SARS-CoV-2 spike protein to host cell receptor ACE2, stop viral entry into the host cell and block viral RNA replication. Also, saikosaponins (A, B2, C, and D), triterpene glycosides, which are isolated from medicinal plants exert antiviral action against HCoV-22E9, and *Houttuynia cordata* water extract has antiviral effects on SARS-CoV. Moreover, eucalyptus oil has promising potential for COVID-19 prevention and treatment. There is an urgent need for research to improve the function of the human immune system all over the world. As a result, actions for better understanding and improving the human immune system are critical steps toward mitigating risks and negative outcomes. These approaches will be strongly recommended for future emerging viruses and pathogens.

## Introduction

 The immune system consists of a collection of cells, chemicals and processes that protect various organs of the body from foreign antigens, cancer cells, and toxins. On the other hand, defects in one arm of the immune system resulting in an inappropriate response.^1^

 Cytokines are tiny glycoproteins generated by nearly every cell to control the immune response. They are classified according to the basis from which they are formed into pro-inflammatory cytokines (cytokines that alert the immune system due to the existence of infection and threat), anti-inflammatory cytokines (cytokines that induce feedback inhibition and return to an inactive non-inflammatory state and negatively control the inflammatory process), lymphokines (cytokines that are released by lymphocytes and adjust the immune response), chemokine (cytokines that recruit leukocytes to the site of infection or injury) and growth factor ( cytokines which aid the cell to be alive and cause structural modifications in the airways).^2^

 Cytokines are essential in the adaptation of the inflammatory response to self-or not-self hazard molecules. For now, inflammation is a vital defense mechanism for health.^3^ Usually, the severe response is recognized by a rapid start and short periods. This response is supplemented by the stimulation of macrophages and liberation of cytokines like interleukin (IL)-1, tumor necrosis factor-α (TNF-α) and IL-6 that act on fibroblasts and endothelial cells and stimulate an increase in the vascular permeability of the cells. This cytokine also induces adhesion molecules expression that binds to different cells like lymphocytes, neutrophils and monocytes and enables these cells to infiltrate through the walls of the blood vessels to the tissue spaces and fight injury or infection. However, prolonged persistence of an antigen or uncontrolled acute inflammation which fails to end tissue injury may lead to chronic inflammatory response and enhance staffing of many cells, improving the release of pro-inflammatory cytokines and finally increased the microbicidal activity.^4^

 The levels of produced cytokine determine the intensity of the inflammatory response. The pro-inflammatory cytokines are very important mediators to set up an anti-infectious response but an exacerbated production of these cytokines may be deleterious and lead to multi-organ failure and death. In addition to that, anti-inflammatory cytokines are suitable to control the cascade of pro-inflammatory cytokines but excessive production is associated with severe immune suppression.^5^ One of the life-threatening states is the cytokine storm that occurs when the level of circulating pro-inflammatory cytokines suddenly rises, causing a massive influx of lymphocytes into the infected area, and causes disruption of the vascular barrier, multi-organ failure, and, eventually, death.^6^

 The acute to chronic progress should be prevented; the inflammatory response must be depressed to avoid the extra tissue damage. Stopping inflammation is a well-managed process involving the production of control mediators such as cytokine and chemokine. According to that, circulating white blood cells decrease recruitment to injury sites as well as induce a reduction in neutrophil infiltration and apoptosis of spent neutrophils, ultimately causing reverse-regulation of chemokine and cytokines.^7^ In this review, we explained the various types of natural immunomodulators, and their effect on cytokine storm syndrome, and the management of severe acute respiratory syndrome coronavirus 2 (SARS-CoV-2).

## Immunological features of COVID-19 associated cytokine storm

**Figure 1:**
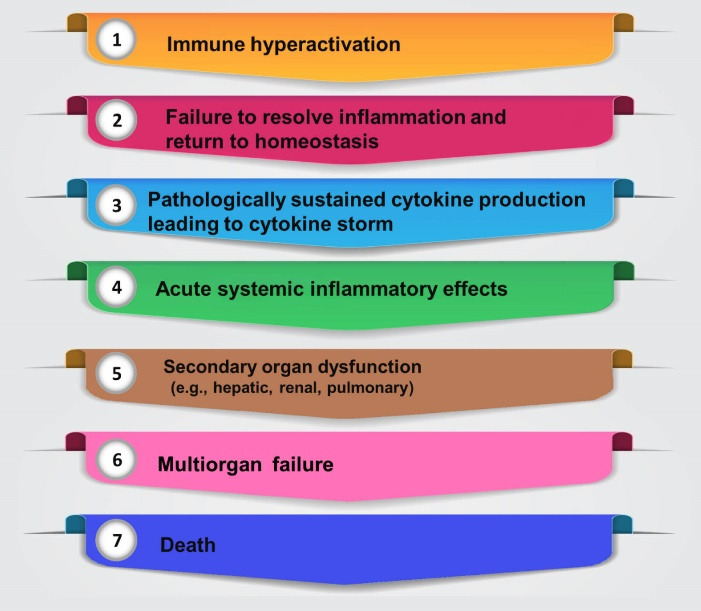
Immunological features of COVID-19 associated cytokine storm.

 Coronavirus is a single-stranded RNA (+ssRNA), and enveloped that belongs to Coronaviridae. Many species of the CoV family infect mammals and birds, causing gastrointestinal infections and upper respiratory tract. It mostly induces the common cold in humans, but complications such as pneumonia and SARS-CoV-2 can happen.^8^ Most of COVID-19 patients have a high degree of pro-inflammatory response causing the cytokine release syndrome (CRS) with a notable rise in the degree of several pro-and anti-inflammatory cytokines and chemokines, such as interferon (IFN)-α, interferon-gamma induced protein 10 (IP-10), IL-1ra, IL-2ra, IL-6, IL-10, IL-18, hepatocyte growth factor (HGF) and monocyte chemotactic protein-3 (MCP-3). The pro-inflammatory effect of IL-6, which is a multifunctional cytokine, plays a role in the inflammatory storm’s modulation. The main functions of IL-6 are the activation of T cells and the promotion of B differentiation and antibody formation (Figure 1).^9^

 Immunomodulation of the immune system with different natural products or drugs can restore the immune system and give some help in reversing the angry of lymphocytes and targeting cytokine to increase the chance of survival.

## Natural products as immune system modulators

 Natural products have a well-known therapeutic efficacy such as the metabolic disorders, cardiovascular diseases, inflammation, and viral infection. Herbal medicines, fatty acids, and probiotics are examples of natural products that play a role in the immune function. They regulate the immune system in a pleiotropic mode, and take a part in diverse adaptive and innate immunity processes. So, natural products have efficacy for directed immune modulators, in the management of definite kinds of immunological and inflammatory diseases, like prevention of Crohn’s disease, ulcerative plaque psoriasis and rheumatoid arthritis, colitis, among other immunological and inflammatory diseases.^10^

###  Polyphenols

 Polyphenols are active natural products familiar with immunomodulatory activities. They control immunity by affecting the immune cell regulation, pro-inflammatory cytokines’ synthesis, and gene expression. They deactivate NF-κB (nuclear factor kappa-light-chain-enhancer of activated B cells) and change mitogen-activated protein kinase (MAPK) and arachidonic acids pathways. Polyphenols are common in plants, since they are products of secondary metabolism and can be found as glycosides or free aglycones.^11^ The polyphenol family has thousands of structural deviations (over 8000), and in vivo and vitro studies have shown that plant polyphenols have anti-inflammatory properties, indicating that they could be used as therapeutic implements for a variety of acute and chronic diseases.^10,12^ Furthermore, polyphenols’ capability to modulate the development of many pro-inflammatory genes and the immune system contributes to the regulation of inflammatory signaling.^13,14^ In vivo and in vitro reports, the murine and rat macrophages exhibited the resveratrol inhibited cyclooxygenase (COX), peroxisome proliferator-activated receptor-gamma (PPAR-γ), and stimulated endothelial nitric oxide synthase (eNOS).^15,16^

 Curcumin and its chemical analogues cause inhibition of the of NF-κB stimulation by many diverse inflammatory stimuli.^17^ In the system of human cells, the curcumin and analogues decreased the expression of inflammatory cytokines such as tumour necrosis factor (TNF) and intercellular adhesion molecule-1 (ICAM-1), and vascular cell adhesion molecule-1 (VCAM-1).

 Wang et al stated that the TNF-α and IL-6 secretion is reduced by polyphenols extracted from chamomile, and quercetin without 1L-1β alteration.^18^ A polyphenol-rich diet can help patients with COVID-19 to decrease inflammation due to the hyper-activation of cytokines as TNF-α, IL-1β, IL-6, and IL-8.^19^ In general, polyphenols modulate inflammatory cytokines, which is one of the most common mechanisms by which they use immune-modulator effects.

**Figure 2:**
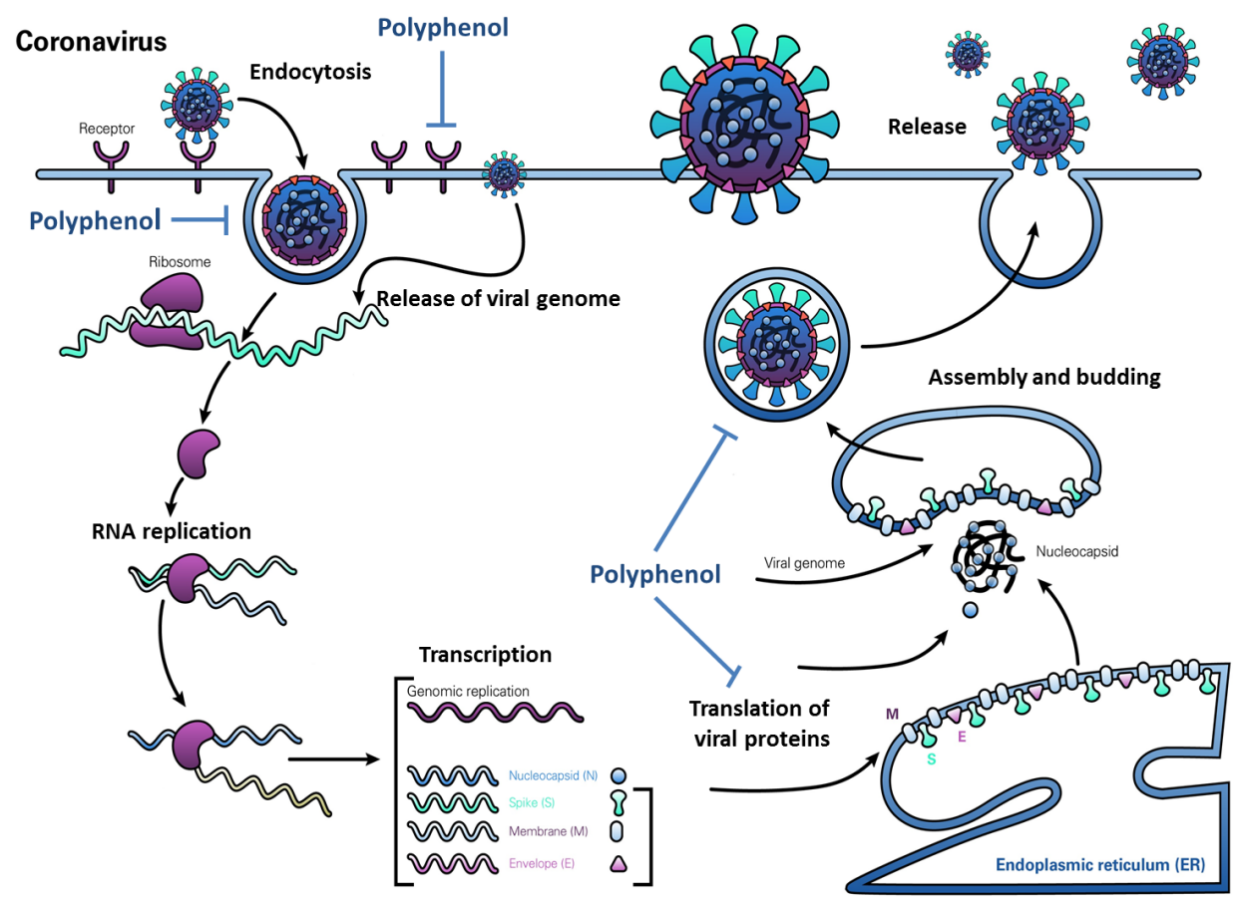
Polyphenols effect on various steps of the SARS-CoV-2 life cycle. Polyphenols potentially block the binding of SARS-CoV-2 spike protein to host cell receptor ACE2, stop viral entry into the host cell, and block viral RNA replication and protein processing

 Diagram show polyphenols Effect on various steps of the SARS-CoV-2 life cycle. Polyphenols potentially block the binding of SARS-CoV-2 spike protein to host cell receptor angiotensin-converting enzyme 2 (ACE2), stop viral entry into the host cell, and block viral RNA replication and protein processing (Figure 2).

###  Effect of zinc on immunity 

 Zinc is a popular dietary supplement, which is discovered in 1961, and is necessary for human physiology.^20^ Also, it can be used as single or in divided doses, and some formulations are designed to be administered intravenous or intramuscular.^21^ Zinc is prescribed in doses of 25–50 mg/d, and has few side effects. However, the higher doses of 200–400 mg/d cause epigastric pain, vomiting, and fatigue.^22,23^

 Zinc is involved in more than 10% of human proteins synthesis and plays a vital role in the immune function.^24^ Also, it has an anti-inflammatory effect by reducing the production of cytokines and biomarkers of oxidative stresses.^25^

 However, zinc deficiency has an effect on immunity, making you more susceptible to infections and the symptoms of zinc deficiency includes cognitive impairment, growth delay, impaired immunity, loss of appetite, heavy hair loss, impotence, diarrhoea, skin, eye lesions, taste defects and postponed wound healing.^26-29^ There are several literatures about the loss of taste or smell and zinc deficiency, which revealed that was due to the chemotherapy or insufficient nutrients.^30-34^

 Moreover, deficiency of zinc is accompanied by a decrease in antibodies secretion, impaired immune system function as low natural killer cell activity, decreased cytokine production, and decreased chemotaxis and oxidative rupture of neutrophils.^35^ Antiviral properties of zinc are essentially via physical routes like virus attachment, infection, uncoating, and by hindering viral protease and polymerase enzymes.^36^

 Many hypotheses recommended zinc compounds as supporting treatment for elderly people with SARS-CoV-2 infection has an illness like diabetes, obesity, or cardiovascular diseases.^37-40^ A recent study highlighted the role of zinc supplementation in SARS-CoV-2, which has promising antiviral effects but, still lack clinical data.^41^

 Studies on cell cultures have shown that the high concentrations of zinc and the addition of pyrithione to stimulate cellular import of zinc ion will stop the replication of different RNA viruses, including several picornaviruses and influenza viruses.^42,43^ Zinc modulates T-cell functions, and preventing immune system hyper-activation by modulating and balancing cytokines.^44^

 A recent study conducted in the United States using electronic medical records discovered a high recovery rate among patients treated with hydroxychloroquine and azithromycin with zinc sulphate.^45^

###  Lactoferrin as a part of innate immunity

**Figure 3:**
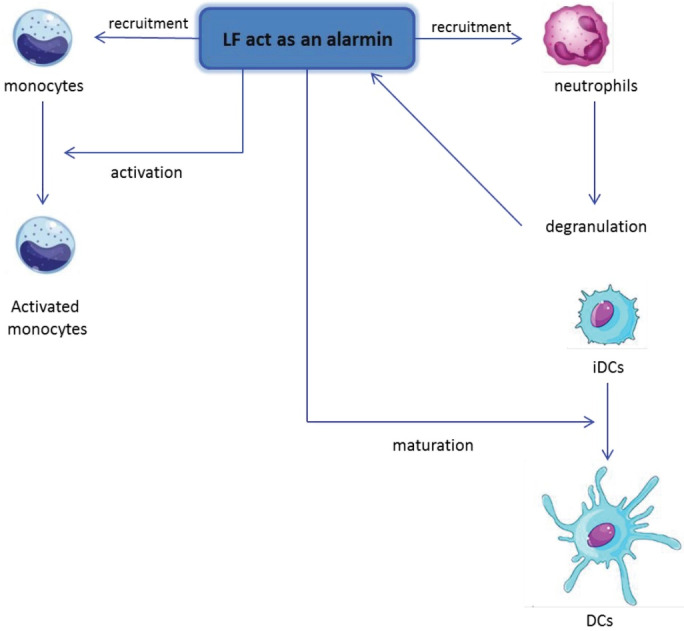
An overview of the main targets of lactoferrin.

 Understanding of lactoferrin antimicrobial efficiency, interaction with host cells, and immune modulation allows this promising protein as a possible antibiotic substitute. Lactoferrin antimicrobial effect directly or indirectly affects bacteria, viruses, and parasites. Lactoferrin can also modulate immune responses by interacting with different lactoferrin targets, like pathogen-associated molecular patterns and their receptors, glycosaminoglycans, and cell receptors. Lactoferrin can also act as an alarmin, allowing the production of neutrophils and monocytes as well as the activation and maturation of monocytes and immature dendritic cells (Figure 3). Lactoferrin can also target other cells, such as epithelial cells, by interacting with the lactoferrin receptor, resulting in uptake and nuclear translocation or transcytosis of these cells.^46^

 Lactoferrin, or lactotransferrin is a globular glycoprotein of the transferrin family found in a variety of secretory fluids, like nasal secretions, milk, saliva, and tears. There is a high concentration of lactoferrin in human colostrum, human, and cow milk. Lactoferrin has antimicrobial activity and is part of the innate immunity, at mucosal surfaces. Lactoferrin exhibits some of its biological functions through interaction with DNA and RNA, polysaccharides, and heparin, to form complexes with them.^47^ Lactoferrin inhibits many human and animal viruses with DNA and RNA genomes, including cytomegalovirus and HIV.^48-52^ Lactoferrin binds to the same lipoproteins as many viruses, expelling the virus particles. Lactoferrin can interact with the cell membrane and bind directly to viral particles as in hepatitis viruses.^53^ Further, an indirect antiviral effect is achieved by affecting natural killer cells, granulocytes, and macrophages that act in the early stages of viral infections.^54^ No direct reaction occurred between host cell angiotensin-converting enzyme 2, which is the functional receptor for SARS-CoV, and SARS-CoV-2 and lactoferrin.^55,56^

 It is widely assumed that lactoferrin can prevent viral entry by interacting with heparan sulphate proteoglycans.^57^ Recently, the study by Zwirzitz et al showed that lactoferrin regulate the activation of the plasminogen, which controls the coagulation stimulated by the virus.^58^ Mesoporous silica nanomaterials have recently attracted researchers’ interest because of their potential biomedical applications, superior biocompatibility, and biodegradability. Furthermore, mesoporous silica nanoparticles are a promising nano-vehicle with high drug loading as well as time-dependent drug release.^59,60^

 Also, tannic acid was used to change the surface of mesoporous silica nanomaterials as a delivery nano-vehicle to accelerate bone healing and regeneration in bone fractures and disorders.^61^ This study of lactoferrin loaded on mesoporous silica could be a promising prospect in the treatment or prevention of SARS-CoV-2.^61^

###  The antivirals and immunomodulators efficacy of medicinal plants and essential oils 

 In addition to traditional therapies that control viral infection, antivirals that are cost- effective and extremely powerful as vaccines are urgently needed. Cheng et al showed that no specific CoV infection therapies are available. As a result, the condition explains the need to develop the right antiviral drugs to combat CoV infection.^62^ Saikosaponins (A, B2, C, and D), triterpene glycosides isolated from medicinal plants such as *Bupleurum *spp*., Scrophularia scorodonia,* and *Heteromorpha*spp*.,* have previously been reported to exert antiviral action against HCoV-22E9.

*Houttuynia cordata* water extract has a number of antiviral effects on SARS-CoV, including prevention of 3CL viral protease and disrupting the activity of RNA-dependent viral RNA polymerase.^63^ According to Li et al, co-challenge with the virus, extracts from *Pyrrosia lingua*, *Lycoris radiata*, *Lindera aggregate*, and *Artemisia annua* extracts effectively suppress the first stage of HCoV-22E9 invasion, including viral penetration and attachment, and induce anti-SARS-CoV effects.^64^ Natural SARS-CoV enzyme inhibitors like 3CL protease and nsP13 helicase have also been reported, including myricetin, scutellarein and *Isatis indigotica* and *Torreya nucifera* phenolic compounds.

 Many viruses are left lacking protective vaccinations and successful antiviral therapies. It seems impossible to end these viral diseases. Natural ingredients, however, are an excellent source of biodiversity for the detection of new antivirals, the discovery of new interactions between structure and operation, and the creation of efficient protective/therapeutic strategies against viral infections.^65^ Because the several findings in this area are still preliminary, further research is encouraged in the characterization of bioactive ingredients, in the description of the underlying pathways, and in the evaluation of the effectiveness and possible use in vivo to help improve successful antiviral therapies. In addition to further trials, the feasibility of combination treatments with other natural substances or traditional medications should be studied in addition to further trials, as multi-target therapies could lead to eliminating the chance of drug-resistant viruses being produced. Natural ingredients, thus believed, will continue to play an important role in the development of antiviral drugs and will contribute to their development. COVID-19 has become a public health issue. Unsurprisingly, considering its high bioavailability against both the SARS-CoV-2 virus and its inflammatory consequences, there are only a few licensed medications available. The production of vaccines is being vigorously studied. However, it will take a year for it to be available to the public. Off-label therapies including dexamethasone, antimalarial (chloroquine/hydroxychloroquine), antivirals (remdesivir), and monoclonal antibodies that inhibit IL-6 receptors (tocilizumab) are used to treat COVID-19 in various combinations.^66^

 Essential oils (EOs) have long been reported to have anti-inflammatory, immunomodulatory, bronchodilator, and antiviral effects for a long time and SARS-CoV-2 virus activity is suggested. For a long time, EOs have been reported to have immunomodulatory, anti-inflammatory, bronchodilator, and antiviral effects. Clove dried leaves provided 4.8% of the oil for hydrodistillation. The oil study of gas chromatography (GC) and gas chromatography–mass spectrometry (GC-MS) led to the identification of 16 compounds. Eugenol (94.4%) followed by caryophyllene (2.9%) was the main compound.^67^ Recent research performed by Merad and Martin found that there are lung defects in nearly all COVID-19 positive patients. The key causes of disease occurrence and mortality in COVID-19 patients are supposed to be pathological and overactive inflammatory responses to SARS-CoV-2. This hyper-inflammatory syndrome is characterized by an increase in inflammatory cytokines, profound lymphopenia, and significant mononuclear cell penetration in the lungs as well as other organs such as the brain, lymph nodes, spleen, and kidneys. Patients’ systemic cytokine profiles show higher levels of cytokines including IL-7, IL-6, and TNF, as well as a slew of other pro-inflammatory cytokines.^68^

 To investigate the effects of eucalyptol therapies and eucalyptus oils on the recruitment of macrophages and monocytes in response to infections and lung inflammation, many in vitro and ex vivo experiments have been performed. Data from these studies suggest that eucalyptus oil and its active part, eucalyptol, have marked immunomodulatory properties. Both therapies decreased the release of monocytes and macrophages of pro-inflammatory cytokines, but their phagocytic activities were not prevented. Eucalyptol is also a mucolytic and bronchodilator.^69,70^ Oddly, it has also been demonstrated that eucalyptus oil has disinfection properties and prevents virus development on different utensils and filter instruments.^71^ Data from clinical studies suggests that eucalyptus oil and its active component, eucalyptol, have promising therapeutic potential for COVID-19 prevention and treatment. In this respect, however, further studies are critically required.

 Molecular docking strategies were used by Kumar and colleagues to assess the anti-SARS-CoV-2 effectiveness of menthol, eugenol, and carvacrol, the main aspects of EOs, against various target proteins of SARS-CoV-2. SARS-CoV-2 spike enzyme, primary protease (Mpro), human ACE-2 proteins, and RNA-dependent RNA polymerase all have binding affinities, according to docking results. Kumar et al also found that carvacrol has the capacity to inhibit Mpro and thus disrupt viral replication.^72^

 A second notification letter (MARCS-CMS607753) was sent to a company alleging that a substance is known as “Nobel laurel” had immune-boosting and antiviral properties, as well as anti-corona effects. The FDA has also sent requests to other firms containing misleading statements about their diagnostic instruments or other information, with regard to these suppliers. Hypersensitivity reactions are another concern associated with using EOs. EOs such as linalool and pinene are known to cause a large spectrum of respiratory problems in allergic patients, including seasonal asthma and rhinitis.^71,72^

 In African green monkey and human lung cancer cells, chloroquine (CQ) and hydroxychloroquine (HCQ) have been shown to effectively prevent SARS-CoV-2 infection by blocking viral genome release.^73^ Furthermore, CQ is thought to increase endosomal pH, blocking endosomal maturation and, as a result, the failure to transport and release SARS-CoV-2. In patients with COVID-19, HCQ was also shown to suppress the development of inflammatory cytokines.^74^

 Around the globe, the sunflower, *Helianthus annuus L*, has a variety of nutritional and medicinal advantages around the world. Antimicrobial, antioxidant, anti-inflammatory, anti-hypertensive, cardiovascular benefits, and wound-healing are given by polyunsaturated fatty acids, flavonoids, phenolic compounds, and vitamins.^75^ In ethnomedicine, it is being used to cure a series of diseases, including cardiovascular disease, bronchial, laryngeal, and respiratory illnesses, cough, cold, and whooping cough.^76^

 Antioxidants such as phenolic acids, flavonoids, vitamins, and trace metals are abundant in the edible seeds and sprouts. Flavonoids are phenolic compounds found in a range of vascular plants that have antiallergic antimicrobial, anti-inflammatory, antiviral, anti-thrombotic, and vasodilator properties.^77,78^

 Tocopherols and vitamin E are the main constituents of sunflower oil. Tocopherols are fat-soluble antioxidant vitamins that can be used in both vivo and vitro.^79^ Sunflower seeds and sprouts are high in niacin, as well as vitamins A, B, and C. Minerals such as iron, calcium, magnesium, arsenic, potassium, selenium, and zinc are abundant.^80^ Enzymes (glutathione dehydrogenase, catalase, and guaiacol peroxidase), carotenoids, peptides, and phenolic compounds (flavonoids, tocopherols and phenolic acids) are all products of different antioxidants.^81^ Sunflower oil has anti-inflammatory properties, and it is thought to reduce paw edema caused by carrageenan by 79.5% as compared to indomethacin (56.2%), which is a popular anti-inflammatory product.^82^

*Helianthus annuus* was used as an antimalarial, anti-inflammatory, anti-asthma, antioxidant and antimicrobial agent and antitumor. Numerous chemical compounds have been identified and characterized from different parts of the plant, including sabinene (monoterpene), heliangolides (sesquiterpene lactones), alpha-pinene, helikauranoside (diterpene), and the leaf alkaloid and phenolic group; helianthoside (triterpene) and the flower saponin group; fatty acids, tocopherol, tannins, and polyphenols of the leaf group. According to the findings, 96% of *H. annuus *root ethanol extract has the greatest antimalarial activity as compared to the other components. It was found to have more efficacy as a therapeutic antimalarial drug than a preventative antimalarial drug. Similarly, as it comes to the antimalarial mechanism of inhibiting blood detoxification, the 96% ethanol extract of *H. annuus* root has the greatest inhibitory effect when compared to the other components of *H. annuus*; the inhibition is also stronger than CQ, a typical medication. As a result, it’s safe to assume that ethanol extract of *H. annuus *root has a possible antimalarial drug, which may be a new target for the development of natural plant-based antimalarial agents.^83^

## Conclusion

 COVID-19 is a serious infection that can cause pneumonia and other complications like sepsis and multi-organ failure. The majority of people with severe COVID-19 were found to have CRS, implying that COVID-19 complications result from a sudden severe immune response to the infection, releasing elevated levels of both pro- and anti-inflammatory cytokines and chemokines. Natural products have always been well-known for their medicinal value in different diseases. With the emergence of COVID-19 and the lack of specific therapies, they have begun to be extensively investigated for their antiviral and immunomodulatory activities Several natural products were found to have immunomodulation activity, anti-oxidant effect as well as an antiviral effect through interference with different stages in the life cycle of SARS-CoV-2. For instance, natural products containing polyphenols, such as chamomile that contains Quercetin, have an anti-inflammatory effect, antioxidant effect as well as antiviral effect. This suggests that a diet rich in polyphenols may help reducing inflammation in patients with COVID-19. A lot of natural products containing EOs were found to have anti-inflammatory, immune-modulatory and antiviral effects. Eucalyptus oil has also been mentioned, as an example of beneficial EOs, for its promising potential for COVID-19 prevention and treatment. The review has also presented the immune-modulatory and antiviral properties of zinc and lactoferrin that led to considering them promising in COVID-19 prevention and treatment.

 In conclusion, natural products serve as excellent source of biodiversity for the development of new antiviral agents. Since a lot of findings in the potential antiviral activity of natural products are still preliminary, the review has highlighted the future perspectives on exploring natural immunomodulators, evaluating their effectiveness and assessing the possibility of their use in the treatment of cytokine storm in COVID-19.

## Author Contributions

**Conceptualization: **Heba Salah Abbas.

**Data curation: **Manal Ahmed Nabil, Heba Salah Abbas.

**Formal analysis: **Manal Ahmed Nabil.

**Investigation:** Mona Mohamed Abd-elhakeem, Rania Mostafa Abd El Galil, Omar Ahmed Reyad, Heba Ahmed Mohamed, Salma Emad Saber Ismail.

**Methodology:** Heba S Abbas, Mona Mohamed Abd-elhakeem, Rania Mostafa Abd Elgalil, Manal Ahmed Nabil.

**Resources:** Heba S Abbas, Mona Mohamed Abd-elhakeem, Rania Mostafa Abd Elgalil, Manal Ahmed Nabil, Omar Ahmed Reyad, Heba Ahmed Mohamed, Salma Emad Saber Ismail.

**Supervision:** Heba Salah Abbas.

**Writing original drafts: **Heba Salah Abbas, Mona Mohamed Abd-elhakeem, Rania Mostafa Abd El Galil, Omar Ahmed Reyad, Heba Ahmed Mohamed, Salma Emad Saber Ismail, Manal Ahmed Nabil

**Writing, reviewing and editing:** Heba Salah Abbas, Manal Ahmed Nabil.

## Ethical Issues

 Not applicable.

## Confilict of Interest

 The authors declare that they have no competing interests.
